# Development and initial testing of a GDM information website for multi-ethnic women with GDM

**DOI:** 10.1186/s12884-015-0578-0

**Published:** 2015-07-05

**Authors:** Mary Carolan-Olah, Cheryl Steele, Gillian Krenzin

**Affiliations:** Nursing and Midwifery, College of Health and Biomedicine, Victoria University, St Alban’s Campus, PO Box 14228, Melbourne, 8001 Australia; Western Health, Diabetes Education Service, Western Hospital, Gordon St., Footscray, VIC 3011 Australia

**Keywords:** Gestational diabetes, Self-management, Knowledge, Intervention, Internet

## Abstract

**Background:**

Gestational diabetes mellitus (GDM) affects approximately 5–15 % of pregnant women in Australia. Highest rates are seen among women who are obese, from specific ethnic backgrounds and low socio-economic circumstance. These features also impact on uptake of self-management recommendations. GDM that is not well managed can give rise to serious pregnancy complications. The aim of this project was to develop and test an intervention to improve knowledge of GDM and GDM self-management principles.

**Methods:**

A web-based intervention, consisting of resources aimed at a low level of literacy, was developed and tested among multi-ethnic women at a metropolitan hospital in Melbourne Australia. A basic one-group pre-test/post-test design was used to explore the impact of the intervention on knowledge, in 3 domains: (1) Knowledge of GDM; (2) food values, and;(3) GDM self-management principles. Questionnaire data was analysed using Statistical Package for the Social Sciences (SPSS), version 21.0. Fisher’s exact test was used to test for an improvement in each knowledge scale.

**Results:**

Twenty-one women with GDM, from multi-ethnic backgrounds, participated in the testing of the intervention. Results indicated that the intervention was effective at improving knowledge scores and this effect was greatest in the first domain, knowledge of GDM. Although some improvement of knowledge scores occurred in the other two domains, food values and self-management principles, these improvements were less than expected. This finding may relate to a number of misunderstandings in the interpretation of the web resource and survey questions. These issues will need to be resolved prior to proceeding to a clinical trial.

**Conclusion:**

Initial results from this study look promising and suggest that with some improvements, the intervention could prove a useful adjunct support for women with GDM from multi-ethnic and low socio-economic backgrounds. Conducting a randomised controlled trial is feasible in the future and will provide a useful means of examining efficacy of the intervention.

## Background

In Australia, Gestational Diabetes Mellitus (GDM) affects approximately 5 % of pregnant women overall [1] and figures are considerably higher among women from specific ethnic backgrounds, such as Asian and South Asian. In such groups, GDM rates may be 2–3 times higher than national rates [2, 3]. These high rates are a concern as GDM is linked to a number of adverse outcomes for mothers and infants. Mothers are more likely to experience: induction of labour; pre-term birth, caesarean section, hypertension and extended hospital stay [1]. Infants of mothers with GDM are predisposed to hypoglycaemia [4], higher rates of stillbirth, macrosomia and birth injury [5], low apgar scores, admission to special care nursery, and longer hospital stays [1, 4]. Careful management of GDM reduces these risks [4, 6].

Self-care is the most usual approach to GDM management and women are taught to: monitor their blood glucose levels; adjust their dietary intake to GDM recommendations, and; increase physical activity, in a bid to maintain blood glucose levels within normal limits. These changes can be very difficult and, for some women quite extensive, due to previous dietary and exercise habits. For these reasons, many women struggle to adhere to GDM recommendations [7–10], particularly as changes must be adopted immediately [8, 11]. Additionally, in order to successfully self-manage the condition, women need to understand GDM, the importance of self-management [4], what constitutes a healthy diet, the impact of different foods on blood glucose levels, and the amount and type of exercise to undertake [8]. Emotional support is critical to motivation and a number of studies indicate that such support contributes positively to GDM skills development [7, 10]. Conversely, a number of factors are identified as likely to impact negatively on the adoption of health recommendations. These factors include: low educational attainment [12], limited health literacy [13], and low levels of English language proficiency [14]. Each of these features increase the likelihood that women will experience significant difficulty self-managing their GDM.

Overall, there is a need for GDM interventions that target low income, ethnically diverse and low literacy groups, as these groups are at high risk of developing GDM. However, despite this clear need, such programs are rare, and only two published studies were identified that reported on interventions to improve levels of knowledge and GDM outcomes among low socio-economic and minority groups [15, 16]. In the first instance, Hoppichler and Lechleitner [16] tested an intervention, which involved repeated intensive dietary counselling, among locally born women with GDM, compared to low literate migrant Turkish women, also with GDM, in Austria. Secondly, Mendelson et al., [15] used a similar approach for low income Mexican-American women with GDM, in California, USA. Both studies considered their interventions successful and also emphasised the importance of ensuring acceptability of educational resources [15, 16]. Furthermore, the literature suggests that in order for GDM interventions to be effective in low income groups, resources need to be pitched at a low level of health literacy [17] and experts in the field recommend using strategies such as pictorial representation of important information and repetition of simple instructions [18]. The provision of culturally sensitive resources is also recognised as important and as likely to improve acceptability and uptake of the intervention [15, 19].

With this information in mind, the website intervention, reported in this paper, aimed to address such issues as cultural sensitivity, low health literacy and low levels of English proficiency, in the belief that women with GDM need resources that they understand and that are culturally relevant to them. The intervention was developed for use in a low socio-economic area of Melbourne [20], where women incur a higher GDM incidence than that recorded nationally [21]. In this area, there are high levels of obesity [22], and as many as one third of the population speak a language other than English at home [23]. In this paper, development and initial testing of the intervention, is discussed. The intervention was planned to serve as an adjunct to routine educational sessions by the dietician and diabetes educator. The team also aimed to test ease of use, relevance and acceptability of the website for the target group of women, and to consider the feasibility of proceeding to a clinical trial.

### Theoretical model

The philosophy of the intervention was based on empowerment of pregnant women [24, 25] and the website was developed along the principles of adult learning theory, that is, the understanding that adults will dedicate effort to learning when the aims and objectives are realistic and important to them [26]. Moreover, within adult learning theory there is an assumption that adults draw on personal experience to make sense of and to integrate new information [27]. Thus, the intervention has incorporated a series of steps anchored to the women’s previous knowledge of food values and dietary concerns, and includes small incremental changes that are likely to be adopted. This approach acknowledges that pregnant women come from diverse backgrounds with different experiences, motivation and understanding. Another psychological theory that has informed the work, is Leventhal et al.’s common sense theory [28, 29]. Leventhal et al.’s [28] common sense theory suggests that individuals create a mental image of their illness, in this case GDM, based on various forms of information at their disposal. Such information commonly includes lay information, from friends and family, more formal information such as from the doctor or midwife, and the individual’s own sense making of their experiences with the illness [29]. We therefore incorporated a number of images to assist with comprehension of GDM, such as the following image, which diagrammatically represents the basis of the disorder:

One of the difficulties with GDM is a tendency for women to underestimate the seriousness of the condition, based on it’s transient quality and we aimed to address this difficulty by re-iteration of the impact, for the baby, of unmanaged GDM. We choose this approach, as in our earlier study, we found that ‘thinking about the baby’ was a powerful motivator for women to manage their GDM [10].

The overall challenge was to convey the urgency of behavioural change without unnecessarily alarming the women.

## Methods

A basic pre-test/post-test survey questionnaire design was used to measure differences, if any, in knowledge of GDM, food values and GDM self-management principles before and after the intervention. This particular approach was chosen as it is considered suitable for measuring changes in behaviours or attitudes [30] and it has been successfully used in a number of nursing and health related studies [31, 32]. Additionally, we intended this testing of the intervention as a means of identifying any weaknesses in the design, and any deficits in general user-friendliness and acceptability for the target group of women. For these reasons, we included three additional questions in the post-test questionnaire, where participants were asked to give feedback on the intervention, in terms of (1) ease of use; (2) value of the information on the website; and (3) any changes or inclusions they would recommend.

### Data collection

#### Instruments

**Fig. 1:**
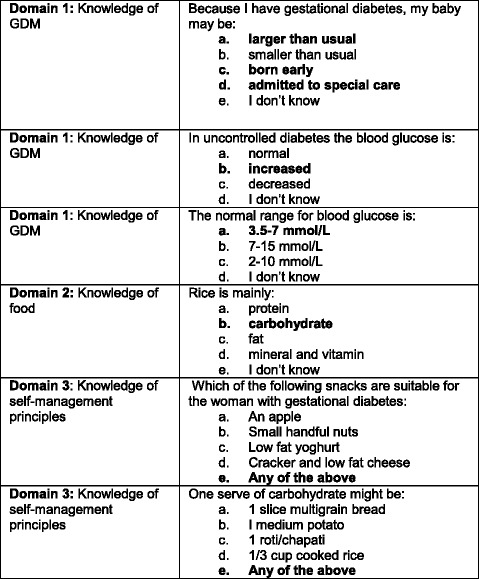
Sample questions

The instrument used was the Knowledge of GDM questionnaire [33] which is based on an earlier validated questionnaire, the Diabetes Knowledge Scale, developed to measure knowledge and understanding of diabetes types 1 and 2, food values, and maintenance of blood glucose levels [34]. A number of modifications were made to the earlier questionnaire in a bid to make it more relevant to GDM and changes were informed by an expert panel, as reported elsewhere [33]. The Knowledge of GDM questionnaire, has now been used successfully among women with GDM from a number of ethnic groups, including Vietnamese, Indian and locally born Australian women [33], and White American, African American and Asian women in America [35]. The questionnaire is composed of twenty-four questions, which fall into three main domains: (1) Knowledge of GDM, including the impact of GDM on mother and baby, and recognising normal glycemic levels; (2) Knowledge of food, including food values; and, (3) Knowledge of GDM self-management principles (see Fig. 1 for sample questions). The majority of questions had one correct answer and were scored simply as correct/ incorrect. Four questions had more than one correct answer, and were scored as correct (all correct answers identified) or incorrect (did not identify all correct answers). The additional 3 questions which sought the women’s views of the website, as above, completed the post-test questionnaire.

### Description of intervention

In earlier studies, we found that most pregnant women in the area, despite their low income status, had access to smart phones and/or computer and that this was their preferred medium for learning about GDM [33]. For these reasons, we developed a web-based intervention, for use on home computers, tablets and smart phones. Women accessing the intervention progress through a series of information sheets, which address topics such as *‘what is GDM’* and ‘*what is healthy eating’*. The intervention also offers practical advice and instruction such as *what to do if you are hungry between meals*. Instructions are phrased simply as short sentences, with a green tick, for acceptable foods, and a red x for poor choices. There are a large number of pictures and minimal amounts of text, and these are recognized as useful approaches for addressing low levels of literacy [36, 37]. An explanation of the amount of exercise that is required, and the type of exercise that is safe in pregnancy, is also included. Representation of ethnic specific food (Western, Vietnamese, Indian, Chinese) and portions appropriate for pregnant women, is placed alongside an explanation of the amount of exercise that is required to burn up the calories contained in the food, as below:

### Recruitment and data collection

A convenience sample of 21 women from the hospital diabetes clinic tested the first version of the intervention. We used the following inclusion criteria:

- Pregnant
- Diagnosis of GDM
- More than 18 years of age
- Singleton pregnancy
- Conversational English, as this initial website is in English

Ethics approval was granted by the Western Health (Sunshine Hospital) low risk ethics panel (HREC/11/WH/81). Women were given a verbal description of the intervention and advised that participation was voluntary. Interested women were invited to go through the website using the touchscreen or computer in the clinic waiting room. Those who agreed to participate, provided written consent. Participants filled in a pre-test questionnaire (approximately 5 min), perused the website while waiting for their clinic appointments, and filled in the post-test questionnaire (approximately 5 min) at their next appointment one week later. A research assistant was available to assist the women to navigate the website and to read out the questionnaire and record the participant’s responses, if the participant wished. Five participants requested this level of assistance.

### Data analysis

A cross table was created comparing the improvement in each of the three scores (GDM, Food, Self-management) against the participant’s level of education (Table 2). Fisher’s Exact test was used to test for an association between each scale’s improvement and the education variable as this test is suitable for small data sets [38]. The *P*-values for these tests are provided below each sub-table.

## Results

### Demographic characteristics

Twenty one women were included in the pre-test/ post-test evaluation. Participants were predominantly from Vietnamese, Australian, Indian and Chinese backgrounds, which is reflective of the population presenting for gestational diabetes services in our region. Age distribution is also typical, with most women aged < 35 years. Completed years of schooling varied from less than 6 years to more than 12 years, with most participants having attended some high school (Table 1).

**Table 1:** Demographic detail

Country of birth	n
Vietnam	6
Australia	5
India	5
China	3
Philippines	1
Sudan	1
Total	21
Age	n
21-25 years	4
26-30 years	6
31-35 years	6
36-40 years	3
41-45 years	1
Not declared	1
Total	21
Years of education	n
<6 (did not complete primary school)	1
11 years or fewer – did not completed high school	10
12 years or more –completed high school	9
Not declared	1
Total	21

A small number of questions were left unanswered, and overall, there were 17 complete sets of GDM and Food improvement scores and 19 complete sets of Self-management improvement scores. Although data analysis suggests insufficient evidence to support an association between education level and improvement of any of the three scales, patterns in the results may suggest otherwise. For example, there were more women who improved in the *11 years or fewer* education group compared to *More than 12 years* (Table 2).

**Table 2:** Knowledge scores by education level

		Education		Total
		11 years or fewer - Not completed high school	More than 12 years - Completed high school	
GDM score change from pre to post*	Improved or stayed at 100 %	7	5	12
		87.5 %	55.6 %	70.6 %
	Stayed the same if below 100 %, or worsened	1	4	5
		12.5 %	44.4 %	29.4 %
	Total	8	9	17
		100.0 %	100.0 %	100.0 %
Food score change from pre to post**	Improved or stayed at 100 %	6	3	9
		66.7 %	33.3 %	50.0 %
	Stayed the same if below 100 %, or worsened	3	6	9
		33.3 %	66.7 %	50.0 %
	Total	9	9	18
		100.0 %	100.0 %	100.0 %
Self-management score change from pre to post***	Improved or stayed at 100 %	5	2	7
		55.6 %	20.0 %	36.8 %
	Stayed the same if below 100 %, or worsened	4	8	12
		44.4 %	80.0 %	63.2 %
	Total	9	10	19
		100.0 %	100.0 %	100.0 %

*Fisher’s Exact test *P*-value for an association between GDM score improvement and Education = 0.294

**Fisher’s Exact test *P*-value for an association between Food score improvement and Education = 0.347

***Fisher’s Exact test *P*-value for an association between Self-management score improvement and Education = 0.170

It was also possible consider the sample as a whole. The percentage of those who improved (or stayed at 100 % correct) post-intervention is given in Table 3, along with its 95 % confidence interval. The 95 % confidence intervals in Table 3 are wide, showing the imprecision of the percentage estimate, in this small sample. Nonetheless, it is clear that the intervention had the greatest impact on the first domain: Knowledge of GDM with 70 % of women improving their knowledge or staying at 100 % post-intervention. The smallest change was in the area of self-management and little more than one third of women improved or stayed at 100 %.

**Table 3:** Knowledge scores composite

	Percentage of sample who improved	95 % Confidence interval
	or stayed at 100 % correct post-intervention	
GDM	70.6 %	(44.0 %, 89.7 %)
Food	50.0 %	(26.0 %, 74.0 %)
Self-management	36.8 %	(16.3 %, 61.6 %)

**Fig. 2:**
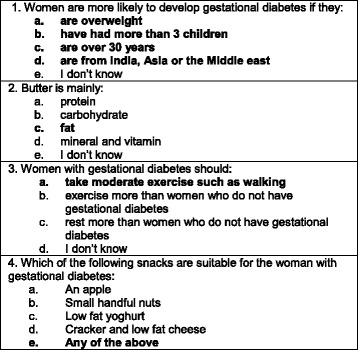
Questions commonly misunderstood

The initial testing identified a number of questions that participants particularly misunderstood (Fig. 2) and which were answered incorrectly more often in the post-test than in the pre-test. A number of factors contributed to this situation. For example, question 1 (Fig. 2) had more than one correct answer and participants were likely to identify being overweight and specific ethnicity as predictors of GDM, but did not recognise maternal age and parity >3, as predictors. For question 2, many women were unable to recognise the food value of butter and very often choose (a.) *protein* or (e.) *I don’t know.* In question 3, participants choose (b.) *should exercise more than women who do not have gestational diabetes* more often than (a.) *should take moderate exercise such as walking* and in question 4, many women identified one or two snacks as suitable for women with GDM, but not all.

In the final group of questions, results indicated that the majority of participants found the intervention acceptable and pertinent to their information needs around GDM (Table 4). Almost all (20/21) found the intervention easy/fairly easy to use and useful/fairly useful. Comments in the final free text question provide some additional insights and participants indicated a number of improvements that they would like, including larger pictures (10 out of the sample of 21 women (10/21), more ethnic specific foods (11/21), less ethnic specific foods (7/21), less text (3/21), more information (6/21), including specifically recipes suitable for GDM. Areas the women identified as particularly useful were the ‘healthy shopping list’ and information about ‘what to do if I am still hungry?

**Table 4:** Ease of use

Easy to use		n	Percent %
	Very easy	18	85.7
	Fairly easy	2	9.5
	Quite difficult	1	4.7
	Total	21	100 %
Useful		n	Percent %
	Very useful	18	85.7
	Fairly useful	2	9.5
	Not very useful	1	4.7
	Total	21	100 %

## Discussion

This paper reports on the development and initial testing of a new intervention, which aims to support women, from multi-ethnic and low health literacy backgrounds, to self-manage their GDM. This intervention has been developed in English initially and there are plans to later amend the program for the Vietnamese community, who have low levels of English language fluency. The aim of this stage of the project was to test the acceptability, ease of use, and usefulness of the intervention for the target population. We also aimed to identify any issues with the website and content and to ascertain both the improvements necessary and the feasibility of proceeding to a clinical trial. Overall, the findings suggest that women found the intervention useful and easy to use. The testing also revealed a number of areas that were effective at promoting greater knowledge scores and a further number of areas that required some improvement. Both of these findings are discussed below.

In the first instance, the intervention proved effective at increasing women’s knowledge scores. The largest improvement occurred in domain 1: Knowledge of GDM and over 70 % of women improved their knowledge scores or stayed at 100 % post-intervention. This is an important effect as knowledge acquisition is established as a necessary first step in understanding and motivation for behavioural change [39]. Moreover, a number of prior studies indicate that women with GDM often complain that the information they receive is insufficient, incomplete or does not meet their needs [11, 33, 40]. Participants in these studies indicate that their efforts to self-manage their GDM are often frustrated by a lack of such knowledge [10, 11]. Feedback in this study suggests that our intervention assists by providing material that participants consider useful and accessible.

In the second domain, knowledge of food, there was a modest improvement in knowledge scores, although the improvement was less than anticipated. This finding may in part be explained by two factors. Firstly, a number of women answered question 2 (Fig. 2, food value of butter) incorrectly, and the percentage getting the question wrong increased considerably in the post-test, which had a negative impact on overall improvement scores. This finding may relate to the fact that many women in the area do not use butter for cooking or food preparation, and therefore may be unfamiliar with this food. However, this explanation does not explain why scores disimproved so markedly from pre-test to post-test. For the future, we plan to simplify and change this question to butter/oil/ghee, which is more in line with local cooking practices. Secondly, a further explanation of repeated incorrect answers about food values may reflect entrenched cultural beliefs about food, and this is something that requires further exploration. In particular, a qualitative exploration of food beliefs and taboos is planned among pregnant women, from specific ethnicities, in our area.

The smallest improvement was recorded in the third domain, knowledge of self-management, where little more than one third of women improved their knowledge scores or remained at 100 %. This finding is in part explained by the misinterpretation of question 3 (Fig. 2), where many women got the answer correct in the first instance and then incorrect in the post-test. Based on this feedback, we plan to amend the text about exercise both in the intervention and in the questionnaire. Participants also got some questions, with more than one correct answer, partially correct, by identifying one variable rather than all correct variables. As these questions required identification of all correct variables to score, this partial knowledge was not recorded. Question 4 (Fig. 2) provides a good example and many women identified one or two healthy snacks but not all four. Nonetheless, these questions provide valuable feedback and allow identification of areas where the required knowledge uptake is insufficient. For example, for question 4, most participants recognise an apple and yoghurt as appropriate snacks, while many do not recognise nuts or crackers and cheese as appropriate. To address this deficit, we may need to further clarify the section, in the intervention, outlining snacks and perhaps add some additional ethnic specific snacks to the questionnaire.

Greater improvement of scores were seen among women with lower rather than higher educational levels. It is not immediately clear what this finding means, however, one possible explanation is that women of lower educational levels may be the most likely to benefit from this intervention, as they are the most likely to have low levels of health literacy [41]. They may also have limited access or ability to decipher health information [42]. Thus, the intervention may offer more support for learning for women with lower educational levels compared to higher levels.

In the literature, fostering knowledge and beliefs, is seen as a necessary first step to health related behavioural change [39] and self-management skill development [43]. This approach is also considered to be in line with adult learning theory [39], where the individual is empowered and motivated by understanding the importance of change [26]. Similarly, a number of studies have found that women are highly motivated to adopt healthy behaviours, in pregnancy to protect the unborn baby [44]. This effect may be even stronger during GDM self-management, if women fully understand the implications of GDM for the child’s future health [8, 45]. Additionally, knowledge of food values, and the basic constituents of a healthy diet, have been positively associated with good nutritional practices, such as eating sufficient fruit and vegetables [46, 47]. Worsley suggests that education may play a part in effecting such behavioural change by encouraging a different set of beliefs about food ([46], p. 583).

However, caution is also advised and a number of studies suggest that knowledge of prudent health behaviours alone, is not sufficient to bring about behavioural change [9, 46, 48]. These studies recognise that dietary changes are difficult and impacted by many factors such as food preferences, cravings and social eating [9] and socio-economic circumstance, food habits and food availability [48].

### Strengths and limitations

The aim of this basic one-group pre-test/post-test study was to explore the impact of the newly developed intervention on knowledge scores in three domains: Knowledge of GDM; food values, and GDM self-management principles. The study has been successful in that it has provided useful feedback that will enable the researchers to improve the intervention. In particular, we plan to clarify the content, amend inconsistencies and ambiguities and add additional information where indicated. These efforts will lead to an improvement of the intervention and a tailoring of the intervention to the needs of the target population. Nonetheless, although the study has achieved the stated aims, some limitations are also present and the small sample size has limited the value of statistical evaluation. For example, p values are very large and although differences in knowledge scores are evident, a larger sample size is required in order to be confident that the associations observed are not just chance. Similarly, there were more women who improved in the *11 years or fewer* education group compared to *more than 12 years* and the meaning of this finding might also be clearer in a larger sample. Finally, only women who spoke conversational English were eligible to participate in this initial testing of the intervention, and this is a limitation that we aim to address in the next phase of the project.

### Implications for practice

The development and testing of the intervention described here has a number of important implications for practice. In the first instance, the demographic profile of GDM, with increased risk factors for multi-ethnic, low socio-economic and poorly educated women, highlights the need for culturally acceptable educational resources pitched at a low level of literacy. Addressing this need is very important as rates of GDM are continuing to increase dramatically in these groups with potentially devastating consequences for mothers and infants and multi-generational effects for families.

## Conclusion

In conclusion, the intervention was effective at improving knowledge scores for women with GDM in the three tested domains: Knowledge of GDM; knowledge of food; and, knowledge of GDM self-management principles, and also that the intervention is a useful adjunct to the routine education sessions women with GDM attend, in our area. Testing has revealed a number of issues, such as ambiguity of information around physical exercise in GDM and some foods. These issues require attention prior to proceeding to a trial.
